# *Fgf9* and *Wnt4* Act as Antagonistic Signals to Regulate Mammalian Sex Determination

**DOI:** 10.1371/journal.pbio.0040187

**Published:** 2006-05-23

**Authors:** Yuna Kim, Akio Kobayashi, Ryohei Sekido, Leo DiNapoli, Jennifer Brennan, Marie-Christine Chaboissier, Francis Poulat, Richard R Behringer, Robin Lovell-Badge, Blanche Capel

**Affiliations:** **1**Department of Cell Biology, Duke University Medical Center, Durham, North Carolina, United States of America; **2**Department of Molecular Genetics, University of Texas M. D. Anderson Cancer Center, Houston, Texas, United States of America; **3**Division of Developmental Genetics, MRC National Institute for Medical Research, London, United Kingdom; **4**INSERM U470, Centre de Biochimie, Parc Valrose, Nice, France; **5**Institut de Génétique Humaine, CNRS UPR1142, Montpellier, France; Osaka UniversityJapan

## Abstract

The genes encoding members of the wingless-related MMTV integration site (WNT) and fibroblast growth factor (FGF) families coordinate growth, morphogenesis, and differentiation in many fields of cells during development. In the mouse, *Fgf9* and *Wnt4* are expressed in gonads of both sexes prior to sex determination. Loss of *Fgf9* leads to XY sex reversal, whereas loss of *Wnt4* results in partial testis development in XX gonads. However, the relationship between these signals and the male sex-determining gene, *Sry,* was unknown. We show through gain- and loss-of-function experiments that fibroblast growth factor 9 (FGF9) and WNT4 act as opposing signals to regulate sex determination. In the mouse XY gonad, *Sry* normally initiates a feed-forward loop between *Sox9* and *Fgf9,* which up-regulates *Fgf9* and represses *Wnt4* to establish the testis pathway. Surprisingly, loss of *Wnt4* in XX gonads is sufficient to up-regulate *Fgf9* and *Sox9* in the absence of *Sry*. These data suggest that the fate of the gonad is controlled by antagonism between *Fgf9* and *Wnt4*. The role of the male sex-determining switch— *Sry* in the case of mammals—is to tip the balance between these underlying patterning signals. In principle, sex determination in other vertebrates may operate through any switch that introduces an imbalance between these two signaling pathways.

## Abstract 2

Adoption of male- and female-specific differentiation events in the gonads is controlled via mutual antagonism between *Fgf9* and *Wnt4*; *Sry* may tip the balance to allow for differentiation down the male pathway.

## Introduction

The development of sexually dimorphic reproductive organs is a common feature among animal species. The testis and ovary represent two divergent pathways of development from the bipotential embryonic gonad. The switch that initiates divergent development of the gonad is highly diverse among species; however, the underlying mechanisms that lead to the establishment of ovary or testis pathways are likely to be conserved. In all species, the embryonic gonad is made up of a mixed population of germ cells and somatic cells. This tissue is remarkable in that all of its cells are believed to be bipotential, and can differentiate into ovarian or testicular lineages [ 1, 2]. Consistent with the idea that cells in this primordium are poised between two developmental pathways, some of the genes that are involved in establishing sexual dimorphism, including *Dax1 (dosage-sensitive sex reversal-congenital adrenal hypoplasia critical region on the X chromosome protein 1), Sox9 (Sry-like HMG box 9), Fgf9 (fibroblast growth factor 9),* and *Wnt4 (wingless-related MMTV integration site 4),* are initially expressed in similar patterns in XX and XY gonads [ 3– 8]. The conventional view of mammalian sex determination is that the basic pathway of organ development is ovarian, and that the testis-determining gene operates by diverting this program toward testis development by simultaneously influencing the fate of the key supporting cell lineage and initiating a male-specific morphogenetic program. All of the experimental evidence suggests that these two processes are closely interwoven. For example, both proliferation [ 9] and migration of cells to trigger testis cord formation [ 10, 11] appear to be closely integrated with Sertoli cell differentiation.

*Sry,* a Y chromosome-linked gene, is the primary sex-determining gene in mammals [ 12– 14]. In the absence of *Sry* expression—in XX embryos, or in XY embryos carrying a deletion of *Sry*—cells in the gonad follow an ovarian differentiation pathway. Genetic evidence from chimeric mice [ 15], and expression studies using reporter transgenes [ 2, 16], indicate that *Sry* expression is required only in precursors of the somatic supporting cell lineage. Expression of *Sry* in these bipotential cells leads to their differentiation as testis-specific Sertoli cells rather than as follicle cells, the parallel cell type of the ovary [ 2]. It is believed that the Sertoli cell is the first cell type to differentiate in the gonad [ 17]. There is substantial evidence that a critical threshold number of Sertoli cells is required to establish testis differentiation [ 9, 15, 18– 20]. In cases where this threshold is not reached, ovary differentiation ensues.

Once *Sry* expression begins, expression patterns of other genes in the gonad begin to diverge. The first gene downstream of *Sry* known to show male-specific up-regulation in Sertoli cell precursors is a related gene expressed in many tissues in the developing embryo *, Sox9*. Disruption of *Sox9* expression in the XY gonad causes male-to-female sex reversal [ 21, 22], whereas increasing the dose of *Sox9* in the XX gonad leads to testis development [ 23– 25]. These studies indicate that *Sox9* plays a central role in sex determination. Unlike *Sry,* which is specific to mammals, expression of *Sox9* is known to be conserved in the gonad of many species. In mammals, *Sox9* is up-regulated immediately after *Sry* expression initiates. Experiments tracing *Sry-*expressing cells using a stable reporter demonstrated that once testis differentiation is established, all Sertoli cells that express *Sox9* are descendants of cells that have expressed *Sry* [ 16], suggesting that activation of *Sox9* is a cell-autonomous effect of *Sry*. However, mutations in several signaling pathways including *Fgf9* and *Igf1r/Irr/Ir (insulin-like growth factor 1 receptor/insulin receptor-related receptor/insulin receptor)* resulted in loss of *Sox9* expression and partial or complete sex reversal [ 26, 27], suggesting that extracellular signaling pathways play a significant role during primary sex determination.

Mice homozygous for a null mutation in *Fgf9* display male-to-female sex reversal caused by disruption of all testis-specific cellular events, including cell proliferation, mesonephric cell migration, testis cord formation, and the differentiation of Sertoli cells [ 26, 27]. *Fgf9,* like many of the founding signals in the gonads, is initially expressed in gonads of both sexes, but becomes male-specific after *Sry* is expressed. In a reciprocal manner, expression of *Wnt4,* which is also initially common to gonads of both sexes, becomes female-specific [ 8]. XX gonads with a null mutation in *Wnt4* display some obvious aspects of testicular differentiation [ 28]. Based on the theory that Sertoli cells initiate all downstream testicular differentiation, this might imply that Sertoli differentiation had been initiated in *Wnt4* ^−/−^ XX gonads. However, expression of Sertoli cell markers was not previously detected in these mutants during fetal stages [ 8, 29], leading to the conclusion that *Wnt4* was not involved in primary sex determination in the gonad.

To integrate these findings, we investigated the genetic relationship of *Sry, Sox9, Fgf9,* and *Wnt4* in the regulatory network that governs the gonadal field. We show that the loss of *Fgf9* in homozygous mutant XY gonads does not affect the expression of SRY or the initial up-regulation of SOX9; however, SOX9 expression is not maintained in the *Fgf9* ^−/−^ mutant gonads, and testis differentiation is aborted. We also demonstrate that FGF9 represses the ovary-promoting gene, *Wnt4.* We hypothesize that FGF9 functions in a feed-forward loop to expand Sertoli precursor cells, which secrete FGF9, to a critical threshold number sufficient to suppress *Wnt4.* This directly or indirectly stabilizes SOX9 expression and secures the male fate of the gonad. WNT4 seems to oppose the male pathway by repressing expression of SOX9 and FGF9. Surprisingly, both male pathway genes are transiently activated in *Wnt4* ^−/−^ XX gonads in the absence of the Y-linked gene *Sry*. Based on this genetic and in vitro data, we suggest that the plasticity of the bipotential gonad is controlled by mutually antagonistic signals between FGF9 and WNT4 in the gonadal field. These signals coordinate sexually dimorphic patterns of growth, morphogenesis, and cellular differentiation.

## Results

### Early Bipotential Expression of FGF9 Resolves to an XY-Specific Pattern by 12.5 dpc

**Figure 1:**
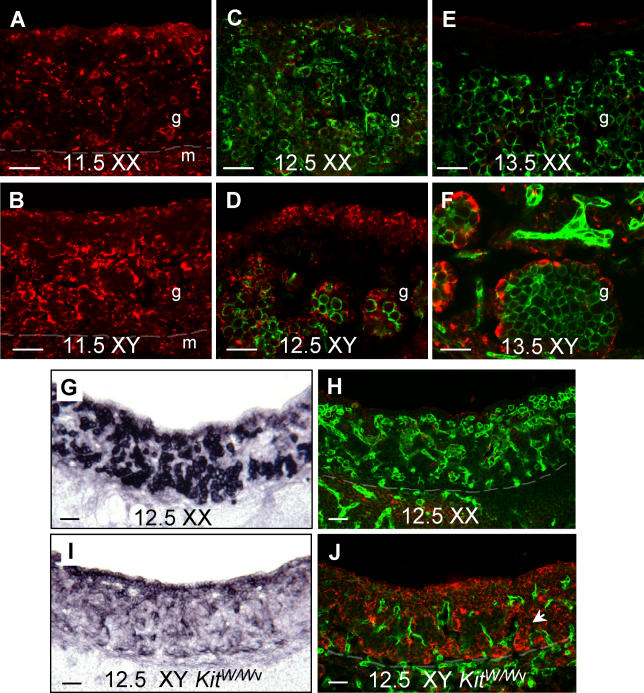
Stage- and Cell-Specific Expression of FGF9 in Embryonic Gonads (A–F) Detection of FGF9 protein (red) at different stages of gonad development. FGF9 is up-regulated in XY gonads at 11.5 (B), 12.5 (D), and 13.5 dpc (F) while it is down-regulated in XX after 11.5 dpc (A, C, and E). No signal was detected in XY *Fgf9* ^−/−^ gonads (unpublished data). (G–J) Serial sections of wild-type XX and compound heterozygous *Kit* ^W/Wv^ XY gonads stained for alkaline phosphatase (purple; G and I) and FGF9 (red; H and J). Testis cords are formed in the absence of germ cells in XY *Kit* ^W/Wv^ mutant gonads at 12.5 dpc (arrowhead in J). Expression of FGF9 is present in the mutant gonads where Sertoli cells are the only remaining cell type in the cords (J). Semitransparent dotted line indicates the boundary between gonad and mesonephroi. PECAM (green) marks germ cells and vascular endothelial cells (C–F, H, and J). The scale bars represent 25 μm. g, gonad; m, mesonephroi.

Using an antibody specific to FGF9, we examined expression during normal gonad development. FGF9 protein was distributed throughout the 11.5 dpc gonad in both sexes ( Figure 1A and 1B). However, by 12.5 dpc, FGF9 was detected only in XY gonads in two domains: in cells near the surface of gonads and in cells located within testis cords. This sex-specific expression pattern was maintained in gonads at 13.5 dpc ( Figure 1C– 1F). FGF9 expression within testis cords was localized to Sertoli cells and excluded from germ cells based on the germ cell membrane marker platelet/endothelial cell adhesion molecule (PECAM) ( Figure 1D and 1F). To confirm this result, we examined agametic gonads from *Kit* ^W/Wv^ compound heterozygous embryos, which develop testis cords containing Sertoli cells only. FGF9 was detectable at normal levels in testis cords in XY *Kit* ^W/Wv^ gonads, where alkaline phosphatase staining verified the absence of germ cells ( Figure 1G– 1J), indicating that FGF9 is expressed by Sertoli cells, and that its expression is not dependent on the presence of germ cells. In summary, FGF9 expression was present in both XX and XY gonads at bipotential stages, and became restricted to XY gonads as testis differentiation proceeded.

### *Sry* Expression Is Normal in Homozygous Null *Fgf9* XY Gonads

**Figure 2:**
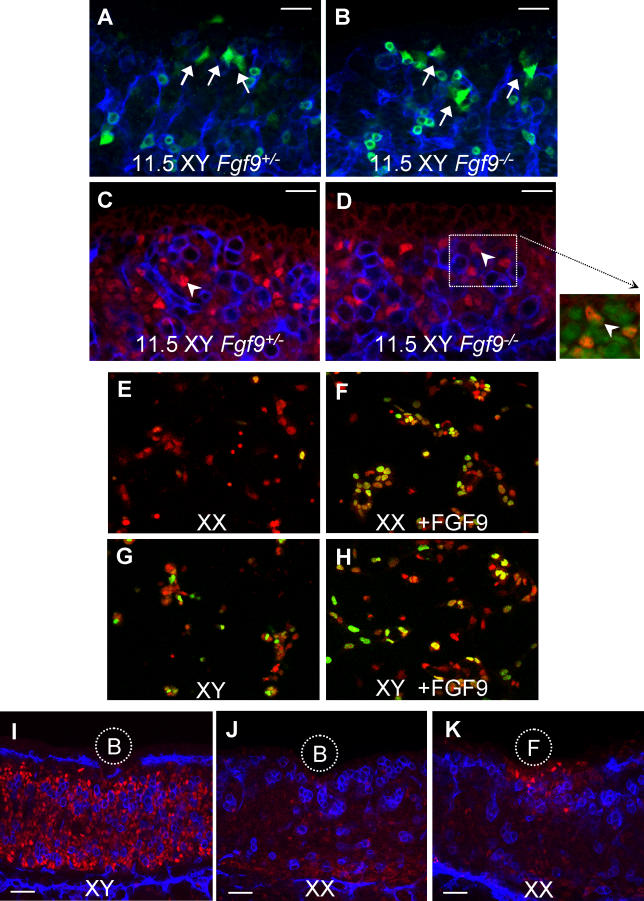
Epistatic Relationship of *Sry, Fgf9,* and *Sox9* (A–D) *Sry* expression is not dependent on *Fgf9*. *Fgf9* ^+/−^ (A) and *Fgf9* ^−/−^ (B) XY gonads at 11.5 dpc expressing GFP (green) from the *Sry* promoter (polygonal cells, arrows). Blood cells show background fluorescence (doughnut-shaped cells). *Fgf9* ^+/−^ (C) and *Fgf9* ^−/−^ (D) XY gonads at 11.5 dpc expressing SRY ^MYC^ protein (red, arrowheads). Inset shows nuclear counterstain (green, Syto13) colocalizing with SRY ^MYC^. PECAM (blue) marks endothelial and germ cells. Scale bars represent 25 μm. (E–K) Exogenous FGF9 can up-regulate SOX9 expression in XX gonads. Immunostaining of SOX9 (green) in primary cultures of gonadal cells. XX cells (E) and XY cells (G) cultured with exogenous FGF9 show induction of SOX9 expression (F and H, respectively). Cells were counterstained using the nuclear marker, Syto13 (red). Immunostaining of SOX9 (red) in gonad explants cultured with BSA- or FGF9-coated beads. SOX9 is expressed in XY gonads and cells contacting FGF9-coated beads (dotted circle labeled “F”) in XX gonads (I and K) but not in XX cells contacting BSA-coated control beads (“B”) (J). PECAM (blue) marks endothelial and germ cells. Scale bars (I–K) represent 50 μm.

The early expression of *Fgf9* in bipotential gonads raised the question of whether *Fgf9* is an upstream regulator of *Sry.* To investigate this possibility, we mated *Fgf9* ^+/−^ mice with a transgenic reporter line that carries an enhanced green fluorescent protein (EGFP) transgene driven by the *Sry* promoter *Sry*- *EGFP*. At early stages, this transgene represents the pattern of endogenous *Sry* expression [ 2]. The expression of the EGFP reporter was detected in *Fgf9* ^−/−^ XY gonads comparable to *Fgf9* ^+/−^ littermate controls ( Figure 2A and 2B), suggesting that transcriptional regulation of *Sry* is independent of *Fgf9*. Sex reversal is caused not only by the loss of normal levels of *Sry* expression [ 30, 31] *,* but also by mutations disrupting SRY import into the nucleus [ 32]. The transgene, *Sry-EGFP,* does not reflect the intracellular distribution of the SRY protein. To investigate this aspect of SRY regulation, we bred *Fgf9* ^+/−^ mice with another *Sry* reporter mouse line carrying a *Myc*-tagged *Sry* transgene, *Sry* ^Myc^, which recapitulates the endogenous intracellular SRY expression pattern [ 16]. Using an antibody against c-MYC, the expression and nuclear localization of SRY ^MYC^ in *Sry* ^Myc^; *Fgf9* ^−/−^ gonads was indistinguishable from littermate controls ( Figure 2C and 2D). These data using two different *Sry* transgenic reporter lines provide evidence that *Sry* expression is not dependent on *Fgf9*. Therefore, *Fgf9* signaling must act in parallel and/or downstream of *Sry* to regulate testis development.

### FGF9 Can Up-Regulate SOX9 Expression

In our previous study we did not observe SOX9 expression at 12.5 dpc in *Fgf9* ^−/−^ XY gonads that fail to develop into normal testes [ 26]. However, the loss of SOX9 expression at 12.5 dpc could be a consequence of the loss of Sertoli differentiation rather than a reflection of the genetic interaction between FGF9 and SOX9. Normally, *Sox9* is weakly expressed in wild-type genital ridges of both XX and XY embryos at 10.5 dpc and, after the onset of *Sry* expression, is up-regulated in XY gonads [ 5, 33, 16]. As results indicated that *Fgf9* functioned downstream of, or in parallel with *Sry,* we investigated whether *Fgf9* was involved in the up-regulation of *Sox9* expression. Primary cell culture and gonad culture systems were used to assess *Sox9* activation by exogenous FGF9. For in vitro cell culture, cells were isolated from 11.5 dpc gonads free of mesonephroi, and cultured on extracellular matrix-coated coverslips with or without addition of purified FGF9 in culture media. After culture for 24 h, SOX9 expression was monitored by an antibody specific to SOX9 in XX cells and control XY cells. Exogenous FGF9 increased cell number in XX and XY cell cultures compared with cells in a duplicate culture without FGF9 treatment (unpublished data), and caused the up-regulation of SOX9 in XX cells ( Figure 2E– 2H). Up-regulation of SOX9 had not previously been seen in whole XX gonads cultured with exogenous FGF9 [ 9]. To explain the difference between experimental results from dissociated XX gonadal cells and XX gonads, we reasoned that the local concentrations of FGF9 might not be high enough to override blocking signals in the intact XX gonad, or that active FGF9 was not efficiently localized or presented in the extracellular matrix of the XX gonad. To test the local effect of FGF9, we modified the XX gonad culture by stably immobilizing FGF9, or BSA as a control, on beads ( Figure 2I– 2K). Under these conditions, SOX9 expression was up-regulated locally in cells near the surface of the XX gonad in contact with the FGF9 bead ( Figure 2K). Taken together, these in vitro data demonstrate that ectopic FGF9 signaling can induce SOX9 expression in XX gonadal cells, suggesting a positive interaction between *Fgf9* and *Sox9*.

### *Fgf9* Is Required for Maintaining SOX9 Expression in XY Gonads

**Figure 3:**
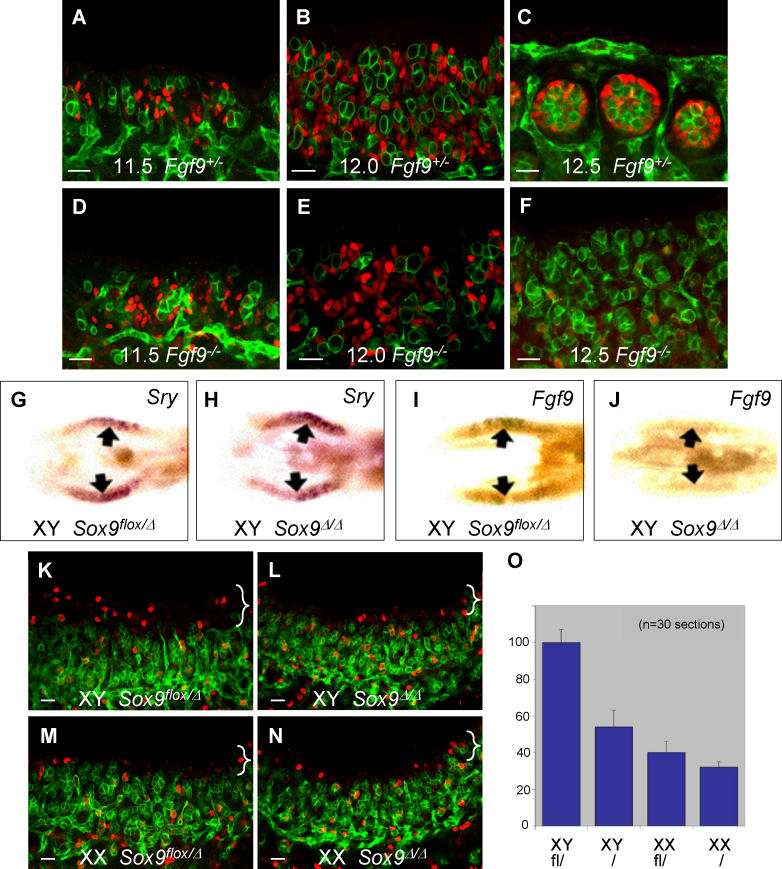
Interdependent Relationship between *Fgf9* and *Sox9* (A–F) Immunostaining of SOX9 (red) in *Fgf9* ^+/−^ and *Fgf9* ^−/−^ XY gonads shows that *Fgf9* is required for maintenance of SOX9. The up-regulation of SOX9 in Sertoli precursor cells appears normal in *Fgf9* ^−/−^ gonads at 11.5 dpc (D) compared with heterozygous littermate controls (A). However, SOX9 is detected in fewer cells in mutant gonads at 12.0 dpc (B and E), and is lost by 12.5 dpc (C and F). (G–J) mRNA whole-mount in situ hybridization for *Sry* and *Fgf9* in *Sox9* ^flox/Δ^ and *Sox9* ^Δ/Δ^ XY gonads shows that *Sox9* is required for *Fgf9* expression. *Sry* expression is detected in both *Sox9* ^flox/Δ^ and *Sox9* ^Δ/Δ^ gonads at 11.5 dpc (G and H), whereas *Fgf9* expression is markedly decreased or absent in *Sox9* ^Δ/Δ^ gonads at 11.5 dpc (I and J). (K–O) Comparison of cell proliferation in *Sox9* ^Δ/Δ^ versus *Sox9* ^flox/Δ^ gonads at 11.5 dpc using immunostaining for phosphorylated histone H3. XY-specific proliferation at the gonad surface (K) is reduced in the absence of *Sox9* (L). Bar graph (O) shows quantitation of proliferation obtained by counting positive cells in the cortical region of each gonad (right brace) and normalizing to the number obtained from XY *Sox9* ^flox/Δ^ gonads. *n* = 30, with five sections of each gonad and three pairs of gonads for each genotype. PECAM, green (A–F and K–N). The scale bars represent 25 μm.

To investigate whether *Fgf9* is essential for the up-regulation of *Sox9* in vivo, we assessed SOX9 expression in loss-of-function *Fgf9* ^+/−^ and *Fgf9* ^−/−^ XY gonads at 11.5–12.5 dpc ( Figure 3A– 3F). In wild-type and heterozygous mutant XY gonads at 11.5 dpc, SOX9 was detected in a small number of cells in the gonad ( Figure 3A). Over the next 6 h of development, nuclear SOX9 accumulated rapidly in cells toward the cortex and the anterior and posterior poles of the gonad, replicating patterns previously reported for both *Sry* and *Sox9* expression [ 2, 16, 34– 36]. This unique pattern was also observed in *Fgf9* ^−/−^ XY gonads ( Figure 3D and 3E). Somatic cells within *Fgf9* ^−/−^ gonads were positive for SOX9 at 11.5 dpc, the earliest stages examined, demonstrating that initial expression and up-regulation of SOX9 were not disrupted in *Fgf9* ^−/−^ mutant XY gonads prior to 12.0 dpc. Notably, in *Fgf9* ^−/−^ XY gonads, SOX9 was no longer detectable by 12.5 dpc, and Sertoli precursor cells never began to organize into normal testis cord structures ( Figure 3F). These data indicate that although *Fgf9* is not required for the up-regulation of *Sox9* in vivo, it is indispensable to maintain *Sox9* expression in Sertoli precursor cells.

### *Sox9* Is Required for *Fgf9* Up-Regulation in XY Gonads

We hypothesized that if the linear relationship among the three genes were *Sry* → *Fgf9* → *Sox9,* expression of *Fgf9* would be normal in XY gonads in the absence of *Sox9*. Alternatively, if the relationship were *Sry* → *Sox9* → *Fgf9,* expression of *Fgf9* should be reduced or absent in XY gonads in the absence of *Sox9*. We examined *Fgf9* expression in *Sox9* homozygous mutant ( *Sox9* ^Δ/Δ^) XY gonads generated by crossing mice homozygous for a conditional null (flox) allele of *Sox9* ( *Sox9* ^flox/flox^) with mice carrying germline-specific *Cre* transgenes, *Prm1-Cre* in male and *Zp3-Cre* in female [ 22, 37]. The *Sox9* null mutant embryos die after 11.5 dpc because of cardiovascular defects [ 37]. Chaboissier et al. [ 22] successfully cultured 11.5 dpc *Sox9* null mutant gonads in vitro and detected male and female markers after 2–3 d of culture, suggesting that *Sox9* mutant gonads are viable and developmentally competent at 11.5 dpc—the time point at which we collected samples to perform mRNA in situ hybridization ( Figure 3G– 3J). The expression of *Fgf9* was significantly decreased or absent in XY *Sox9* ^Δ/Δ^ gonads at 11.5 dpc ( Figure 3J), while *Sry* expression was similar to wild-type ( Figure 3G and 3H), as previously reported [ 20], suggesting that *Fgf9* expression in wild-type XY gonads is dependent on the expression of *Sox9*. These findings also indicate that expression of *Sry* is not sufficient to regulate *Fgf9* in the absence of *Sox9*. Therefore, we conclude that *Sox9* is essential for *Fgf9* expression, and *Fgf9,* in return, maintains *Sox9* expression, generating a positive feed-forward loop between these two genes in XY gonads.

### Like *Fgf9* Mutant Gonads, *Sox9* ^Δ/Δ^ XY Gonads Show Defects in Cell Proliferation

We previously reported that XY-specific cell proliferation is defective in *Fgf9* ^−/−^ XY gonads [ 7]. Because *Sox9* acts as a positive regulator of *Fgf9* expression, we questioned whether cell proliferation in XY gonads was also compromised by the loss of *Sox9*. We examined proliferation in *Sox9* ^Δ/Δ^ gonads at 11.5 dpc using a mitotic cell marker, phosphorylated histone H3. Proliferating cells were more abundant and concentrated in a domain near the surface of wild-type XY gonads, and this XY-specific cell proliferation was evident in XY *Sox9* ^flox/Δ^ littermate controls ( Figure 3K). However, in *Sox9* ^Δ/Δ^ XY gonads proliferation was reduced and similar to XX gonads ( Figure 3L– 3O). This result supports the idea that there is a mutual interdependence between *Sox9* and *Fgf9* generating a positive feed-forward loop, and that both genes are required for the expansion of somatic cells, including Sertoli cell precursors, in XY gonads.

### The Male Pathway Is Aborted in *Fgf9* ^−/−^ Sertoli Precursors

**Figure 4:**
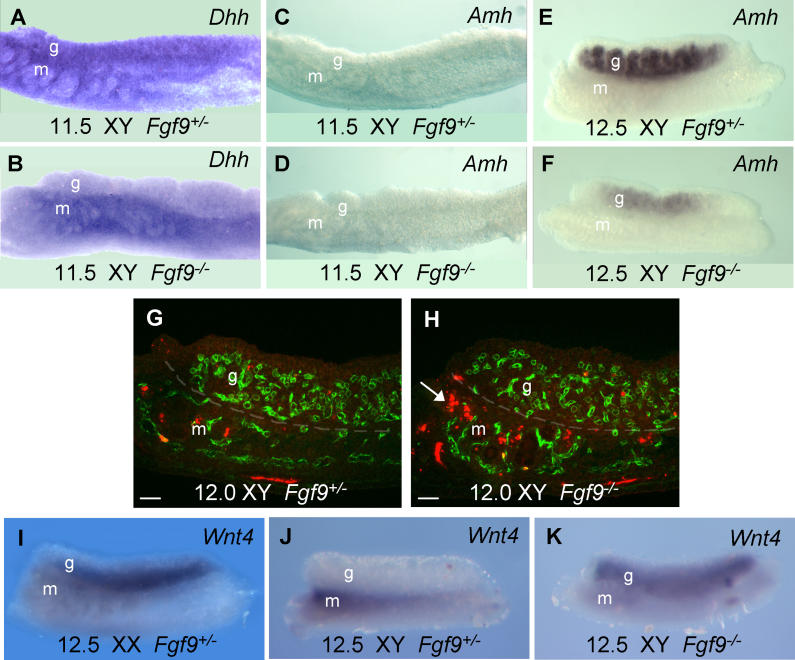
Sertoli Cell Precursors Switch from Expression of Male to Female Pathway Genes (A–F) Whole-mount in situ hybridization for genes in the male pathway downstream of *Sox9, Dhh,* and *Amh*. *Dhh* expression is disrupted in XY *Fgf9* ^−/−^ gonads (g) at 11.5 dpc (A and B). *Amh* expression is severely reduced in XY *Fgf9* ^−/−^ gonads at 12.5 dpc (E and F). (G and H) Analysis of cell death in *Fgf9* ^−/−^ XY gonads using an apoptotic marker, active caspase-3 (red). No increased apoptosis is observed in XY *Fgf9* ^−/−^ gonads (g) compared with control XY gonads, although apoptotic cells are increased around mesonephric tubules (m) of the mutant gonads (arrow in H). Semitransparent dotted line indicates boundary between mesonephros and gonad. (PECAM, green). (I–K) Whole-mount in situ hybridization for *Wnt4,* an ovary marker. *Wnt4* is expressed in *Fgf9* ^−/−^ XY gonads at 12.5 dpc (K) similar to the level in XX *Fgf9* ^+/−^ controls (I) but not in XY controls (J). The scale bars represent 50 μm. g, gonad; m, mesonephros.

Based on the fact that *Sox9* is initially expressed in *Fgf9* ^−/−^ gonads, we investigated whether other genes in the male pathway are activated. We examined two markers for Sertoli cell differentiation, *anti-Mullerian hormone (Amh)* [ 38] and *Desert hedgehog (Dhh)* [ 39] in *Fgf9* ^−/−^ XY gonads using whole-mount in situ hybridization. *Dhh,* which is expressed in XY wild-type and heterozygous gonads beginning at 11.5 dpc, was absent from *Fgf9* ^−/−^ XY gonads, although mesonephric expression was still detected ( Figure 4A and 4B). *Amh,* which is a direct transcriptional target of SOX9 activated after 11.5 dpc [ 38, 40], was detected at reduced levels in *Fgf9* ^−/−^ XY gonads at 12.5 dpc ( Figure 4C– 4F). The residual level of *Amh* suggested that the transient expression of SOX9 in *Fgf9* ^−/−^ gonads at 11.5 dpc was sufficient to activate *Amh,* a direct downstream target. However, the absence of *Dhh* indicated that not all Sertoli pathways are initiated.

The initial specification of Sertoli cell precursors was not affected by the loss of *Fgf9,* as evidenced by normal *Sry* and *Sox9* expression in *Fgf9* ^−/−^ gonads at 11.5 dpc ( Figures 2 and 3D). However, SOX9 expression in XY *Fgf9* ^−/−^ gonads rapidly disappeared ( Figure 3E and 3F), and other Sertoli markers were absent or severely reduced ( Figure 4A– 4F). To investigate the possibility that this loss was due to cell death, we immunostained XY *Fgf9* ^−/−^ gonads at 12.0 dpc—a time point at which SOX9-expressing cells were declining in numbers ( Figure 3E)—for active caspase-3, an apoptotic cell marker. Apoptotic cells were not observed in *Fgf9* ^−/−^ gonads or in littermate controls at 12.0 dpc, although *Fgf9* ^−/−^ samples showed somewhat increased cell death in mesonephric tubules and ducts, another site of *Fgf9* expression ( Figure 4G and 4H). These data suggested that the loss of SOX9 expression in *Fgf9* ^−/−^ XY gonads was not caused by cell death but by the disruption of FGF9/SOX9 feed-forward regulation. To determine whether the aborting of the male pathway in *Fgf9* ^−/−^ Sertoli precursors was associated with the transition of supporting cells from male to female differentiation, we investigated expression of *Wnt4,* an ovary-promoting gene. At 12.5 dpc *Wnt4* was up-regulated in XY *Fgf9* ^−/−^ but not in XY *Fgf9* ^+/−^ gonad controls ( Figure 4I– 4K). This result suggests that *Fgf9* is necessary for the down-regulation of *Wnt4* in differentiating XY gonads at/after bipotential stages.

### *Fgf9* and *Wnt4* Antagonize Each Other

**Figure 5:**
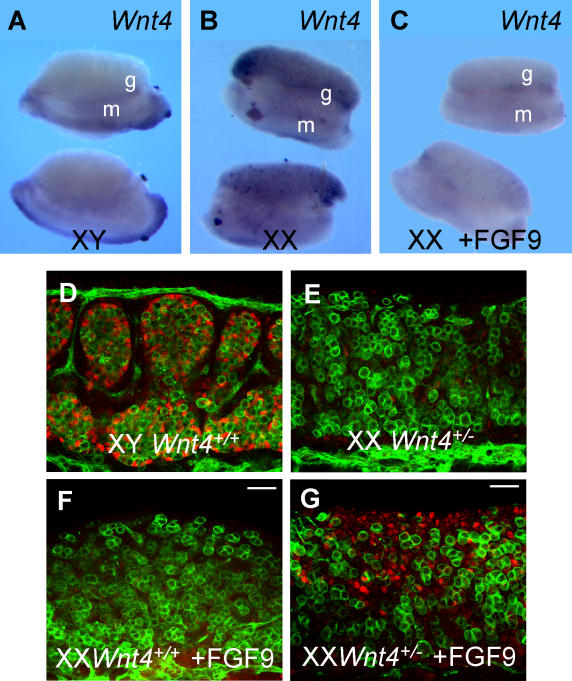
Mutual Antagonism between *Fgf9* and *Wnt4* (A–C) *Wnt4* whole-mount in situ hybridization on gonad cultures. Adding exogenous FGF9 in gonad cultures results in the down-regulation of *Wnt4* expression in cultured XX gonads (C). Controls (A and B) were cultured without FGF9 peptide. (D–G) Reduction in the dose of *Wnt4* allows FGF9 to induce SOX9 in XX gonads. Immunostaining of SOX9 (red) shows that addition of FGF9 up-regulates SOX9 expression in heterozygous *Wnt4* ^+/−^ XX gonads (G), but not in *Wnt4* ^+/+^ XX gonads (F). PECAM, green. The scale bars represent 50 μm.

Our finding that high levels of *Wnt4* persist in *Fgf9* ^−/−^ XY gonads implies a genetic antagonism specifically between *Fgf9* and *Wnt4,* as both SRY and SOX9 are initially expressed in *Fgf9* ^−/−^ XY gonads at 11.5 dpc, yet this is not sufficient to down-regulate *Wnt4.* To test whether exogenous FGF9 could down-regulate expression of *Wnt4,* we cultured the XX gonad/mesonephros complex with or without FGF9 protein, and examined *Wnt4* expression by whole-mount in situ hybridization. Treatment of XX gonads with exogenous FGF9 suppressed the normal expression of *Wnt4* ( Figure 5A– 5C), supporting the hypothesis that *Fgf9,* rather than *Sry* or *Sox9,* functions to down-regulate *Wnt4* in wild-type XY gonads.

**Figure 6:**
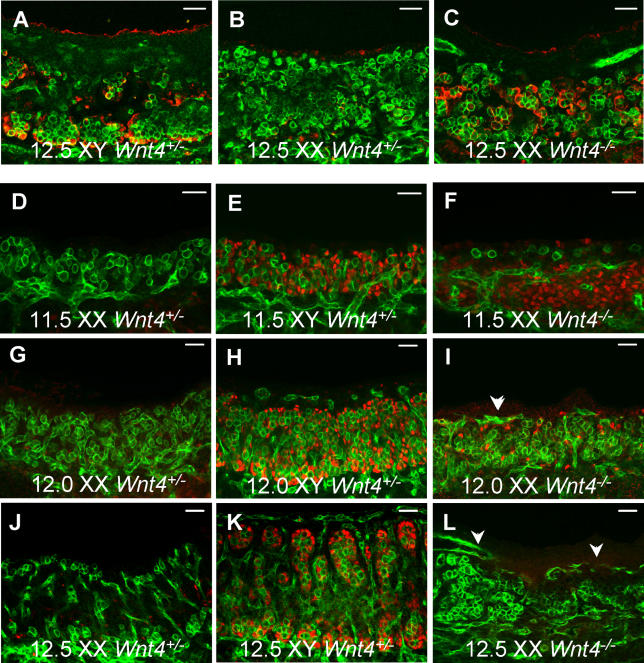
Ectopic Expression of Male Factors, SOX9 and FGF9, in XX *Wnt4* ^−/−^ Gonads (A–C) FGF9 (red) immunostaining shows that FGF9 is expressed in XX *Wnt4* ^−/−^ gonads at 12.5 dpc (C) relative to littermate controls (A and B). (D–L) SOX9 (red) immunostaining shows that SOX9 is transiently up-regulated in XX *Wnt4* ^−/−^ gonads. SOX9 expression is detected in *Wnt4* ^−/−^ XX gonads at 11.5–12.0 dpc (F and I), albeit at reduced levels compared with XY gonad controls (E-H). SOX9 is not detected in control XX *Wnt4* ^+/−^ gonads (D-J) or *Wnt4* ^−/−^ XX gonads at 12.5 dpc (L). The ectopic coelomic vessel in XX *Wnt4* ^−/−^ gonads [ 28] is indicated by arrowheads. PECAM, green. The scale bars represent 50 μm.

We reasoned that if FGF9 and WNT4 do act as opposing signals, then reduction in the dose of *Wnt4* might render the XX gonad more susceptible to the male-promoting effects of exogenous FGF9. To test this possibility, XX *Wnt4* ^+/−^ and *Wnt4* ^+/+^ gonads were cultured in medium with or without FGF9, and were examined for SOX9 expression ( Figure 5D– 5G). We found that FGF9 induced SOX9 up-regulation in XX *Wnt4* ^+/−^ gonads, but not in *Wnt4* ^+/+^ XX gonads ( Figure 5F and 5G). These results demonstrate antagonism between WNT4 and FGF9 under in vitro gain-of-function conditions. To test antagonism between these factors under loss-of-function conditions in vivo, we investigated whether *Fgf9* is derepressed in the absence of *Wnt4* ( Figure 6A– 6C). Using an antibody against FGF9, we found that FGF9 was expressed in *Wnt4* ^−/−^ XX gonads but not in *Wnt4* ^+/−^ XX controls ( Figure 6B and 6C). This result suggested that FGF9 is normally down-regulated by WNT4 in XX gonads.

Given our finding that FGF9, a positive regulator of *Sox9,* is derepressed in XX *Wnt4* ^−/−^ gonads, we asked whether expression of SOX9 might also occur in XX *Wnt4* ^−/−^ gonads ( Figure 6D– 6L). An antibody against SOX9 revealed that expression was initially up-regulated in *Wnt4* ^−/−^ XX gonads at 11.5 dpc ( Figure 6F and 6I), although it was rapidly down-regulated by 12.0 dpc and absent at 12.5 dpc ( Figure 6I and 6L). This finding was confirmed by mRNA in situ hybridization, which also detected *Sox9* transcripts in 11.5 dpc *Wnt4* ^−/−^ XX gonads ( Figure S1). *Wnt4* ^−/−^ XX gonads do not increase in size comparable to normal XY gonads ( Figure 6C, 6F, 6I, and 6L), and Sertoli cell differentiation and testis cord formation do not occur. Nevertheless, it is noteworthy that up-regulation of SOX9 occurs in this case in the absence of *Sry,* by eliminating the antagonistic effect of *Wnt4* and up-regulating FGF9, supporting our hypothesis that sex determination occurs by tipping the balance between these two opposing signals.

## Discussion

Many studies support the view that cells in the undifferentiated gonad are bipotential; the supporting cell precursor lineage can develop into follicle cells or Sertoli cells. In *Fgf9* ^−/−^ XY gonads, cells initially embark on the Sertoli pathway, but in the absence of *Fgf9* can neither maintain *Sox9* expression nor establish downstream male pathways. The loss of Sertoli cells in XY *Fgf9* ^−/−^ gonads is not due to cell death, but instead to a transition of supporting cell fate as SOX9 expression is lost. We suggest that in the absence of the antagonizing activity of FGF9, WNT4 signals predominate and govern somatic cell fate in the gonadal field.

The *Drosophila* genital disk is also a field of cells that normally follows one of two sexually dimorphic fates. For many years it was believed that the fate of each cell in the genital disk was under the cell-autonomous control of *double sex (dsx),* the key regulator of the sex determination pathway. However, mosaic studies have shown that the genetic sex of the cells in the anterior/posterior organizers of the disk, not the sex of the majority of cells in the disk, regulate the sexually dimorphic fate of the disk. This occurs through the sex-specific regulation of WNT, FGF, and transforming growth factor beta signaling, which in turn regulate the growth, cell differentiation, and morphogenesis of the disk [ 41, 42]. Sex-specific regulation of gonad organogenesis in vertebrates may occur in a similar manner, where some cells are cell-autonomously responsive to the sex-determining switch; however, the establishment of the male or female program occurs through the non-cell-autonomous activity of classic signaling pathways that act in an antagonistic manner and coordinate growth, cell differentiation and morphogenesis in the gonad.

The interplay between cell-autonomous and non-cell-autonomous pathways in the mammalian gonad is not well understood. In XX↔XY chimera experiments, XX cells can be recruited to the Sertoli lineage, indicating that non-cell-autonomous signaling mechanisms operate under these conditions [ 15]. Other more recent studies have suggested that paracrine signals could be involved in the establishment of Sertoli cells [ 43– 46]. The current study reveals that ectopic FGF9 can induce SOX9 under conditions in which XX cells are dissociated ( Figure 2F), when an FGF9-coated bead is directly applied to the XX gonad ( Figure 2K), or when the dose of *Wnt4* is reduced ( Figure 5G). Whether FGF9 normally acts non-cell-autonomously in vivo to recruit XY cells to the Sertoli lineage by up-regulating SOX9 is not clear. We show that *Sry* can initially up-regulate SOX9 in the absence of *Fgf9,* suggesting that FGF9 is not necessary for this step. However, FGF9 may act to trigger cell proliferation, increasing the number of Sertoli precursors above a threshold needed to stabilize the male pathway, consistent with threshold requirements deduced from earlier studies using XX↔XY chimeric gonads [ 15]. Since Sertoli cells produce FGF9, loss of proliferation of Sertoli precursors may result in a reduction of the overall level of FGF9, and/or other male paracrine signals, below a critical threshold level required to antagonize the influence of WNT4. This model is appealing, because it links cell proliferation, believed to be required for establishment of the male pathway [ 9], with Sertoli fate determination. A recent study by Yoshioka et al. [ 47] showed that misexpression of *Fgf9* in chick nephrogenous mesenchyme led to the expansion of gonadal marker gene expression, implicating *Fgf9* in gonadal cell proliferation across species.

It has been suggested that SOX9 represses WNT4 based on misexpression studies [ 48]. Here we show that the addition of FGF9 protein to XX gonad explant cultures repressed the expression of *Wnt4*. Down-regulation of *Wnt4* is unlikely to occur through SOX9, as SOX9 is not up-regulated in this situation [ 7]. Furthermore, although both SRY and SOX9 are initially expressed in *Fgf9* ^−/−^ XY gonads, *Wnt4* is not down-regulated in the absence of *Fgf9* ( Figure 4K). These findings support the idea that FGF9 acts as the antagonist of *Wnt4*. Antagonism of WNT signals may be a multistep process involving both the transcriptional down-regulation of *Wnt4* observed in this study and the destabilization of downstream Wnt intracellular pathways that antagonize SOX9 expression, as shown in chondrocyte differentiation [ 49], or that compete for intracellular signal transducers as has been reported in other systems [ 50, 51]. Future work will address these possibilities.

In support of the idea that *Wnt4* antagonizes the male pathway, we found that the loss of *Wnt4* caused the up-regulation of both SOX9 and FGF9 in XX gonads where *Sry* is absent. It appears that the male pathway can be initiated by disrupting the balance between *Wnt4* and *Fgf9,* a finding that has strong implications for other vertebrate sex-determination systems in which *Sry* is not the sex determining factor. However, up-regulation of *Sox9* is not sufficient to establish testis development in this mutant, as occurs in *Odsex* and other gain-of-function mutants where *Sox9* is misexpressed in the XX gonad [ 24, 25]. In those two misexpression cases, *Sox9* expression may have been artificially sustained by exogenous regulatory sequences that bypass the fine dosage balance in this signaling network.

In *Wnt4* mutants, SOX9 expression is not maintained. In light of the observation that the *Wnt4* ^−/−^ XX gonad does not increase significantly in size ( Figure 6), it is possible that the FGF9/SOX9-expressing population did not reach a critical threshold. Alternatively (or in addition), another male-specific factor normally dependent on *Sry* may be required to sustain SOX9 expression, possibly FGF-binding proteins in the extracellular matrix or FGF receptors. It is equally plausible that there are other female-specific factors that antagonize the establishment of SOX9 expression. It has been observed that several other WNTs are expressed in the XX gonad [ 52], and these or other factors may partially compensate for the loss of *Wnt4*.

These findings suggest that WNT4 signaling normally acts as a repressor of the male pathway by interfering with the up-regulation of SOX9 expression. One report of a duplication of the region of human Chromosome 1, which includes *WNT4,* led to an intersex phenotype [ 53]. However, the report constitutes only circumstantial evidence. Such a role is not supported by efforts to misexpress *Wnt4* in XY gonads, which have led to very mild phenotypes with no evidence for defects in Sertoli cell differentiation [ 54]. It is possible that WNT4 protein did not function as an active signal in these transgenic mice, either because it was not expressed in the right cells, at the right time, or at the right level. Consistent with our data and the partially sex-reversed phenotype of *Wnt4* ^−/−^ XX mutants, other WNTs or additional female factors may be required.

**Figure 7:**
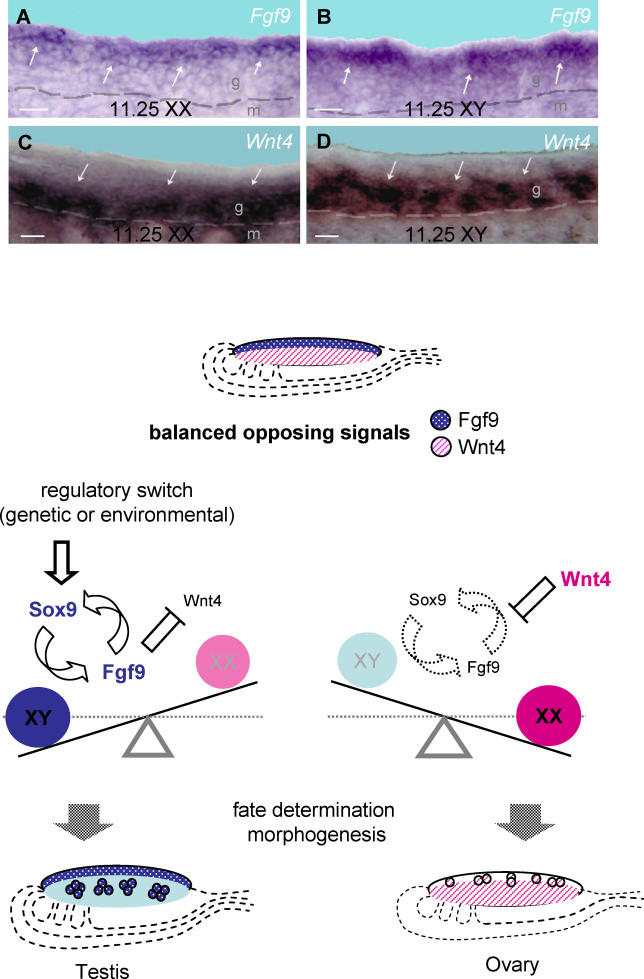
Opposing Signals Regulate Sex Determination in the Bipotential Gonad In both XX and XY gonads at 11.25 dpc (15 tail-somite stage), *Fgf9* transcripts (white arrows) are detected near the gonad surface (A and B), whereas *Wnt4* transcripts are detected near the gonad mesonephric boundary (C and D). We propose a model in which the fate of the gonad is balanced between these competing signals. A genetic or environmental switch initiates the male pathway by creating an imbalance between these signals. In mammals, this imbalance occurs through the up-regulation of *Sox9*. *Sox9* up-regulates *Fgf9,* and *Fgf9* maintains *Sox9,* forming a positive feed-forward loop in XY gonads. In this situation, the balance between FGF9 and WNT4 signals is shifted in favor of FGF9, and the dominance of the male pathway is established. In the absence of a feed-forward loop between SOX9 and FGF9 (e.g., in XX gonads), WNT4 blocks *Fgf9,* initiating the female pathway.

The switch that controls sex determination is biologically diverse. *Sry* is not present in nonmammalian systems; however, antagonistic signaling between FGFs and WNTs may be the conserved mechanism that balances the gonad between testicular and ovarian fates in vertebrates. In theory, any genetic or environmental switch may tip the balance toward the male pathway. Based on our findings we propose that cells in the mammalian gonad are balanced between two competing cell fates by counterbalanced signaling pathways, *Fgf9,* expressed near the coelomic surface, and *Wnt4,* expressed near the mesonephric border ( Figure 7). In mammalian XY gonads, the onset of *Sry* expression initiates the male pathway by up-regulating *Sox9*. SOX9 up-regulates *Fgf9,* which initiates a *Sox9/Fgf9* feed-forward loop that accelerates commitment to the male pathway. In XX gonads or XY mutant gonads lacking *Sry, Sox9,* or *Fgf9,* the SOX9/FGF9 feed-forward loop is not established, and WNT4 gains control of the gonadal field. This results in the down-regulation of *Sox9* and *Fgf9,* tilting the balance toward commitment to the female pathway. Further experiments will be required to define the molecular mechanism of FGF9 and WNT4 action. However, our in vivo and in vitro data strongly support the antagonistic relationship of these two signaling pathways in regulating expression of the testis-determining factor SOX9.

## Materials and Methods

### Animals and genotyping.

The *Fgf9* mutation was maintained on a C57BL/6 (B6) background that leads to sex reversal in 100% of XY *Fgf9* ^−/−^ offspring. *Sry-EGFP* mice, a kind gift from K. Albrecht and E. Eicher, were initially on a mixed B6/129 and were backcrossed to B6 for five generations. Offspring were then crossed to *Fgf9* ^+/−^ and intercrossed and backcrossed to B6 in alternating generations. All XY *Fgf9* ^−/−^ offspring showed complete sex reversal. *Sry* ^Myc^ mice were maintained on a CBA background, and *Wnt4* on a mixed 129/SVJ background. Mutant embryos were sexed by PCR using Y chromosome-specific primers and were genotyped as described [ 2, 16, 26, 55]. Mice homozygous for the *Sox9* deletion were generated using a germline-specific gene deletion system as described [ 20].

### In situ hybridization and immunocytochemistry.

In situ hybridization was performed on paraformaldehyde-fixed/OCT embedded cryosections, as described [ 56]. Whole-mount in situ hybridization was performed as previously described [ 57]. Probes used for in situ hybridization were: *Amh* [ 58], *Dhh* [ 20], *Wnt4* [ 8], and *Fgf9* [ 59]. Digoxigenin-labeled probes were prepared according to the Boehringer-Mannheim-Roche protocol.

Antibodies used in whole-mount immunocytochemistry were: mouse monoclonal anti-N-MYC (Cell Signaling Technology, Beverly, Massachusetts, United States; 1:100), rabbit anti-SOX9 (gift of F. Poulat; 1:1,000), rat anti-PECAM (Pharmingen, San Diego, California, United States; 1:500), rabbit anti-caspase-3 fragment (BD Bioscience, San Diego, California, United States; 1:100), and rabbit anti-phosphorylated histone H3 (Cell Signaling; 1:250). Antibody binding was detected using fluorophore-conjugated secondary antibodies (Jackson ImmunoResearch, West Grove, Pennsylvania, United States) as recommended. For FGF9 immunostaining, gonads were prepared as frozen sections, and rabbit anti-mouse FGF9 (Cell Science; 1:50) and anti-rabbit IgG conjugated with peroxidase secondary antibody were used, followed by amplification with tyramide-Cy3 fluorophore (Molecular Probes, Eugene, Oregon, United States). This antibody did not detect FGF9 in 12.5 dpc ovary or *Fgf9* ^−/−^ null mutant gonads. Immunostained samples were mounted in DABCO and imaged on a Zeiss LSM420 confocal microscope.

### Primary gonadal cell culture.

11.5 dpc embryos were collected from CD1 mice, and sex was determined by staining amnions as described [ 15]. Whole genital ridges were dissected and gonads were separated from mesonephroi. The gonads were treated with collagenase (0.025%) and trypsin (0.025%) in HAT buffer at 37 °C for 10 min. After the digestion, cells were mechanically dissociated by pipetting, washed in DMEM, plated on 10-mm diameter coverslips coated with extracellular matrix (Sigma), and were cultured in DMEM containing 5% fetal bovine serum and 1× antibiotics/antimycotics at 37 °C, 5% CO _2_. In one of duplicate cultures, FGF9 (R&D Systems, Minneapolis, Minnesota, United States) was added to the final concentration of 50 ng/ml in the culture medium. After 36 h of culture, cells were fixed and immunostained for SOX9. Syto13 (Molecular Probes) was used for nuclear counterstaining, according to the manufacturer's instruction.

### Gonad explant culture.

Gonad/mesonephros complexes were dissected at 11.5 dpc and cultured on agar blocks for 36 h at 37 °C, 5% CO _2_ as described [ 60]. For FGF9 treatment, 50 ng/ml FGF9 (R&D Systems) was added directly to the culture medium, or an FGF9-loaded bead was placed on the surface of gonad. To coat beads, heparin-agarose beads (Sigma, St. Louis, Missouri, United States) were incubated in 50 μg/ml FGF9 for 2 h, and washed five times in culture medium.

## Supporting Information

Figure S1 Confirmation that Transcription of *Sox9* Is Up-Regulated in *Wnt4* ^−/−^ XX Gonads
Whole-mount in situ hybridization for *Sox9* detected *Sox9* expression in 11.5 dpc *Wnt4* ^+/−^ XY controls and *Wnt4* ^−/−^ XX gonads.
(428 KB PPT)

## Abbreviations

dpc: day post coitum
EGFP: enhanced green fluorescent protein
FGF: fibroblast growth factor
PECAM: platelet/endothelial cell adhesion molecule
SOX: SRY-like HMG box
SRY: sex-determining region of the Y
WNT: wingless-related MMTV integration site
